# Gold Nanoparticles for Brain Tumor Imaging: A Systematic Review

**DOI:** 10.3389/fneur.2018.00328

**Published:** 2018-05-14

**Authors:** Antonio Meola, Jianghong Rao, Navjot Chaudhary, Mayur Sharma, Steven D. Chang

**Affiliations:** ^1^Department of Neurosurgery, Stanford University, Stanford, CA, United States; ^2^Department of Radiology, Stanford University, Stanford, CA, United States; ^3^Department of Neurosurgery, University of Louisville, Louisville, KY, United States

**Keywords:** gold, nanoparticle, brain tumor, Raman scattering, magnetic resonance imaging, photoacoustic imaging, glioma, blood-brain barrier

## Abstract

**Background:**

Demarcation of malignant brain tumor boundaries is critical to achieve complete resection and to improve patient survival. Contrast-enhanced brain magnetic resonance imaging (MRI) is the gold standard for diagnosis and pre-surgical planning, despite limitations of gadolinium (Gd)-based contrast agents to depict tumor margins. Recently, solid metal-based nanoparticles (NPs) have shown potential as diagnostic probes for brain tumors. Gold nanoparticles (GNPs) emerged among those, because of their unique physical and chemical properties and biocompatibility. The aim of the present study is to review the application of GNPs for *in vitro* and *in vivo* brain tumor diagnosis.

**Methods:**

We performed a PubMed search of reports exploring the application of GNPs in the diagnosis of brain tumors in biological models including cells, animals, primates, and humans. The search words were “gold” AND “NP” AND “brain tumor.” Two reviewers performed eligibility assessment independently in an unblinded standardized manner. The following data were extracted from each paper: first author, year of publication, animal/cellular model, GNP geometry, GNP size, GNP coating [i.e., polyethylene glycol (PEG) and Gd], blood-brain barrier (BBB) crossing aids, imaging modalities, and therapeutic agents conjugated to the GNPs.

**Results:**

The PubMed search provided 100 items. A total of 16 studies, published between the 2011 and 2017, were included in our review. No studies on humans were found. Thirteen studies were conducted *in vivo* on rodent models. The most common shape was a nanosphere (12 studies). The size of GNPs ranged between 20 and 120 nm. In eight studies, the GNPs were covered in PEG. The BBB penetration was increased by surface molecules (nine studies) or by means of external energy sources (in two studies). The most commonly used imaging modalities were MRI (four studies), surface-enhanced Raman scattering (three studies), and fluorescent microscopy (three studies). In two studies, the GNPs were conjugated with therapeutic agents.

**Conclusion:**

Experimental studies demonstrated that GNPs might be versatile, persistent, and safe contrast agents for multimodality imaging, thus enhancing the tumor edges pre-, intra-, and post-operatively improving microscopic precision. The diagnostic GNPs might also be used for multiple therapeutic approaches, namely as “theranostic” NPs.

## Introduction

Surgery is the mainstay of primary brain tumor management (1). The goal of image-guided neurosurgery for primary brain tumors is to achieve maximal tumor resection, while minimizing neurological morbidity and mortality related to brain manipulation of cortical and subcortical structures (2, 3). Post-surgical residual enhancing tumor volume is a strong significant negative predictor of patient survival, especially in high-grade gliomas (4). Thus, the delineation of tumor boundaries is crucial for optimizing surgical resection and improving overall survival.

Although brain magnetic resonance imaging (MRI) is still the gold standard for brain tumor diagnosis (5, 6), it is accuracy in delineating brain tumor boundaries is limited by the pharmacological properties of the commonly used gadolinium (Gd)-based contrast agents. Indeed, these agents enhance brain tumor regions where there are major defects and abnormal permeability of the blood–brain barrier (BBB) (7). However, BBB permeability is almost normal at in the most peripheral, yet most actively replicating and invasive portions of high-grade tumors (4). Thus, visualization of tumor edges can be sub-optimal as peripheral tumor debulking may be limited, while a more extensive resection could positively impact patient survival (8). In a similar fashion, low-grade primary brain tumors induce a limited increase in BBB permeability, preventing a clear delineation of tumor edges (9). Thus, there is a need for new imaging techniques, enabling the neurosurgeon to better visualize tumor edges, regardless of BBB permeability and tumor histology and grade.

Recently, solid metal-based nanoparticles (NPs) have shown potential as diagnostic probes for brain tumors (10, 11). Nanotechnology has emerged with the goal of better understanding and manipulating materials in order to create NPs ranging from 1 to 100 nm in diameter (11). The potential of the NPs as diagnostic (and therapeutic) agents in neuro-oncology can be attributed to their chemical and physical properties, including their small size, physiological stability, and biocompatibility (11). Several NPs were tested for brain tumor imaging, including quantum dots, iron oxide NPs, superparamagnetic iron oxide NPs, carbon nanotubes, dendrimers, polyelectrolyte complex NPs, calcium phosphate NPs, perfluorocarbon NPs, and lipid-based NPs (11). Nonetheless, the main limitation for the application of metallic NPs *in vitro* and *in vivo* is cyto-toxicity due to degradation and release of toxic metal ions (12).

Gold nanoparticles (GNPs) were discovered more than one century ago. Given their remarkable biocompatibility, negligible toxicity, high-atomic number, and high-X-ray absorption coefficient, they have received significant interest recently for use in multiple imaging technologies (13). Additionally, GNPs synthesis is technically easy and cost effective (14).

To the best our knowledge, this is the first literature review to explore the application of GNPs in brain tumor diagnosis. We aim to: (1) define the main structural features of GNPs that are critical for their biological, toxic, and physical (radiological) properties; (2) review the radiological techniques that can be used in conjunction with GNPs; and (3) review experimental models for testing GNPs and explore any potential studies in humans.

Future research could include creating new GNPs specifically optimized for particular radiological techniques, as well as for preparing experimental models to test GNPs.

## Materials and Methods

The present review was conducted according to the PRISMA statement criteria (15). The literature search was updated to April 30, 2017. No other temporal limits were applied. The search was open to both *in vitro* and *in vivo* studies. Inclusion criteria included GNPs to diagnose any kind of brain tumor in biological models including cells, animals, primates, and humans. The review included only original papers published in Pubmed-indexed peer-review journals, clearly stating the structural features of the GNPs (listed below), the experimental model/s, and the radiological technique/s applied in conjunction with the GNPs. Exclusion criteria included: papers not describing original research (i.e., reviews, perspectives, letters to the editor, commentaries, and abstracts), papers in languages other than English, description of new chemical or physical properties of GNPs without application of biological models, and papers focusing on nanotechnology but not primarily on brain tumor diagnosis or GNPs. The search was performed using the PubMed database and by scanning reference lists of the resulting articles. The search terms were “gold” AND “NP” AND “brain tumor.” Eligibility assessment was performed independently in an unblinded standardized manner by two reviewers (Antonio Meola and Navjot Chaudhary). Disagreements between reviewers were resolved by consensus. The following data were extracted from each paper: first author, year of publication, animal/cellular model, GNP route of administration, GNP geometry, GNP size, GNP coating and imaging tags, BBB-crossing enhancers, imaging modalities applied in conjunction with the GNPs, and main conclusions of the study. Unfortunately, a quantitative comparison between studies or groups was not possible because of heterogeneity of the biological models and technical discrepancies between different GNP formulations. Therefore, no statistical analysis was performed.

## Results

**Figure 1:**
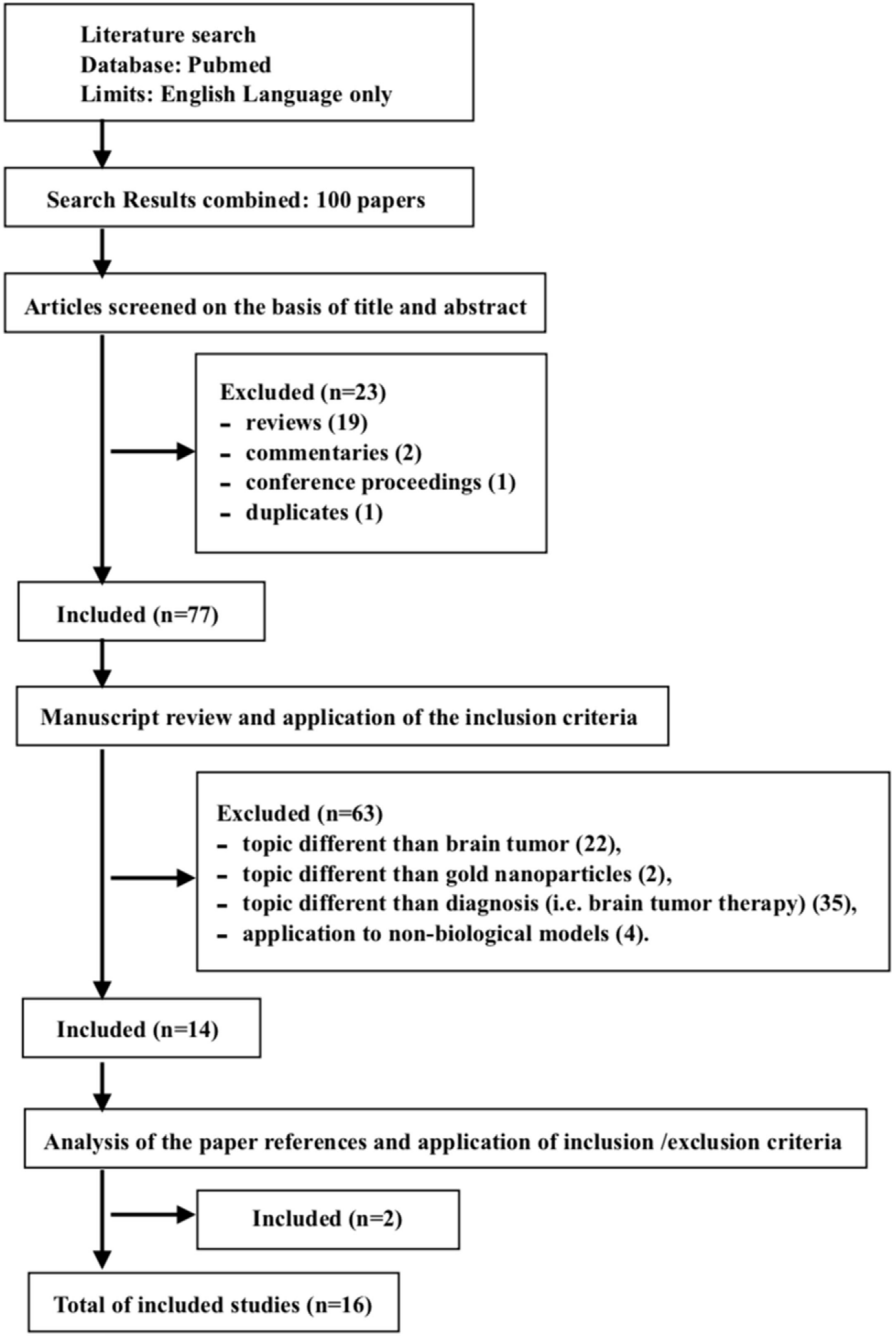
Flow-diagram of study selection, according to PRISMA criteria (15).

A total of 16 studies were included in our review. The PubMed search yielded 100 items. One duplicate was found. Among the collected studies, 85 were discarded because they met the exclusion criteria: reviews (16), commentaries (2), conference proceedings (1), topics different than brain tumor (17), topics different than GNPs (2), topics different than diagnosis (i.e., brain tumor therapy) (18), and applications on non-biological models (4). Two (2) citations (19, 20) were added after reviewing the bibliographies of the included papers (Figure 1). The studies included are summarized in Table 1, and an overview of the main features of the GNPs and of the experimental models is reported in Tables 2–4.

**Table 1:** Overview of the included studies.

Reference	Model	Route of adm.	Shape	Size (nm)	GNP coating and imaging tags	BBB-crossing enhancers	Imaging modality	Main conclusions
Gao et al. (21)	*In vivo*: U87 GBM orthotopic xenograft in nude mice	I.V.	GNS	20	PEG, Gd-DTPA, Raman tag (IR783B), Alkyne/azide group	LRP-1	MRI, SERS microscopy	– The acidic brain tumor environment triggers nanoclustering of alkyne-GNS with azide-GNS, preventing them from returning in the blood stream. – Xenograft edges are persistently enhanced by Gd-DTPA. – Tumor resection is guided by SERS signal
Huang et al. (20)	*In vivo*: RCAS-PDGF/N-tva transgenic mouse model of GBM (overexpression of integrin αvβ3)	I.V.	GNSt/GNS	60	PEG, Raman tag (*N,N*-dimethylformamide) RGDyK/RADyK	RGDyK	SERS microscopy	– RGDyK-GNSts penetrate the GBM significantly better than RADyK-GNSt (non-integrin targeted GNSt). – RGDyK-GNSts define the tumor edges, the local infiltration and satellite foci
Pohlmann et al. (22)	*In vitro*: GS9-6/NOTCH1 + GBM cells	Culture	GNR	50	PVP	No	TEM	– TEM allows visualizing the interaction between cells and GNPs at different GNP concentrations, and between GNPs within the tumor cells
Lai et al. (23)	*In vivo*: GNS-loaded U87 GBM and GBM8401 glioma orthotopic xenograft in mice	Culture	GNS	N/A	Fluorescent tag (MUA)	No	TXM, Fluo	– The GNPs allow tumor localization, visualization of anomalous tumor vasculature and detection of the BBB leakage typical of brain tumors
Kempen et al. (24)	*In vivo*: TS543 GBM xenograft in severe combined immunodeficiency mice	I.V.	GNS	60	Silica shell, Gd, Raman tag *trans*-1,2-bis(4-pyridyl)-ethylene	No	SEM, Optical microscopy	– By complementing the SEM imaging with optical imaging, the GNPs can be identified and localized within the tumor itself
Dixit et al. (25)	*In vitro*: U87 GBM cells, U227 GBM cells. *In vivo*: U87 GBM orthotopic xenograft in mice	Culture/I.V.	GNS	41	PEG, Fluorescent tag (Pc4)	Tf	Fluo	– Tf conjugation significantly enhanced the GNP uptake by GBM orthotopic xenograft with respect to the GNPs non-conjugated with Tf. – Regardless of the Tf conjugation, the GNPs were found to be highly specific for brain tumor tissue, with negligible accumulation in other organs
Dixit et al. (26)	*In vitro*: U87 GBM cells U227 GBM cells. *In vivo*: U87 GBM orthotopic graft in mice	Culture/I.V.	GNS	41	PEG, Fluorescent tag (Pc4)	Tf, FGF	Fluo	– Double-targeted GNPs cross the BBB more efficiently than untargeted GNP-Pc4, leading to higher accumulation levels and to a faster rate of accumulation. – Double-targeted GNPs accumulate in critical organs less than single-targeted GNPs
Cheng et al. (27)	*In vitro*: U87 GBM cells, U251 GBM cells, GBM43 cells, GL261 GBM cells *In vivo*: U87 GBM orthotopic xenograft implanted in athymic nude mice	Culture/I.V.	GNS	21	PEG, Gd, Dox	TAT	MRI	– When compared with Gd-chelates alone, the TAT-GNP-Gd conjugates cause more intense and more lasting enhancement of the brain tumor with signal still detectable after 24 h. – GNPs are washed out from the normal brain within 24 h. – TAT-GNPs conjugated with Dox cross the BBB and are selectively uptaken by tumor cells that are killed. – TAT-GNPs conjugated with Dox significantly increase mice survival with respect to Dox alone or TAT-GNPs alone. – TAT-GNPs conjugated with Dox or with Gd cause no adverse effects *in vivo*
Diaz et al. (28)	*In vitro*: gliosarcoma 9L cells GBM cells, C6 glioma cells, U87 GBM cells, A172 GBM cells, U251 GBM cells, U373 GBM cells, BT2012036 oligodendroglioma, GLINS1 GBM stem cells *In vivo*: GNP-loaded U87 orthotopic xenograft in nude mice; 9L gliosarcoma orthotopic xenograft in mice	Culture (U87 model), I.V or intra-arterial (9L model)	GNS	50/120	PEG, Silica shell, Fluorescent tag (Cyto647) Raman tag (trans-1,2-Bis(4-pyridyl)-ethylene)	MRgFUS, anti-EGFR Ab	MRI, TEM, Fluo, SERS microscopy	– Fluo allows monitoring the growth of GNPs-loaded xenograft. – GNPs cross the BBB in areas treated with MRgFUS. – Anti-EGFR functionalization promotes GNPs uptake by tumor cells. – SERS-active GNPs allow enhancement of the brain tumor edges after MRgFUS *in vivo*
Yuan et al. (29)	*In vivo*: D270 glioma xenograft in mice	I.V.	GNSt	80	PEG	Ultra-short pulsed laser	MPM	– MPM allows micro-angiographic visualization of GNPs in the tumor vasculature. – Low-potency image-guided pulsed laser irradiation allows selective GNSs uptake by the tumor
Schultke et al. (30)	*In vivo*: GNP-loaded C6 glioma xenograft in Wistar rats	Culture	GNS	50	No	No	SynCT	SynCT allows single-cell spatial resolution of GNP-loaded glioma xenograft *ex vivo*
Astolfo et al. (31)	*In vivo*: GNP-loaded F98 glioma xenograft in mice	Culture	GNS	50	No	No	SynCT	– SynCT allows 3D reconstruction and volumetric analysis of GNP-loaded tumor xenograft *in vivo* and *ex vivo*. – Tumor doubling time is assessed by SynCT
Nedosekin et al. (32)	*In vitro*: B16F10 Melanoma cells, MDA-MB-231 Breast cancer cells *In vivo*: MDA-MB-231 Breast cancer xenograft implanted in mice breast	Culture/intratumoral	GNR	<100	PEG	Folate, EpCam, CD 45	PAFC PTC	– Photothermal imaging allows to identify GNRs labeled cancer cells and to detect the intracellular clustering of GNRs. – By applying a laser over the cisterna magna of mice, the GNRs were used to label and detect breast cancer metastasis to the CNS before those became macroscopically evident
Cho et al. (16)	*In vitro*: GNP-loaded U87 glioma cells. *In vivo*: GNP-loaded U87 glioma cell suspension injected subcutaneously in mice	Culture	GNC	50	No	RGDyK (the experiment did not require BBB crossing)	MPM, PAM	– MPM estimates the intracellular uptake of GNCs by glioma cells *in vitro*. – PAM allowed estimating the total number of RADyK-GNCs within the tumor at each time point and quantifying the growth of the tumor
Kircher et al. (19)	*In vitro*: eGFP^+^U87MG cells. *In vivo*: eGFP^+^U87MG xenograft in mice	Culture/I.V.	GNS	60	Silica shell, Gd, Raman tag (*trans*-1,2-bis(4-pyridyl)-ethylene) *Aka* MPR NP	No	MRI, PAM, SERS microscopy	– MPR NP allows MRI, photoacoustic and Raman imaging. – MRI, photoacoustic, and Raman imaging co-localize *in vivo* between them and with histological analysis. – SERS microscopy allowed guiding GBM resection in mice
Noreen et al. (33)	*In vivo*: U87 GBM cells xenograft in mice injected with GPNs	I.V.	GNS	20	No	No	FTIR	– FTIR *in vitro* reveals microvascular architecture of brain tumors (enhanced by BaSO_4_ nanoparticles) and their micro-fenestration (revealed by GNP leak into the extravascular space)

*Anti-EGFR Ab, anti-epidermal growth factor receptor antibody; BBB, blood-brain barrier; Cyto647, cytochrome 647; FGF, fibroblast growth factor; Fluo, fluorescent imaging; FTIR, Fourier-transform infrared imaging; I.V., intravenous; Gd, gadolinium; GNR, gold nanorod; GNS, gold nanosphere; GNSt, gold nanostar; LRP-1, low-density lipoprotein-receptor-related protein-1; MPM, multiphoton microscopy; MPR, magnetic resonance imaging–photoacoustic imaging–Raman imaging; MRgFUS, MR-guided focused ultrasound; MRI, magnetic resonance imaging; MUA, mercapto-urodecanoic acid; PAFC, photoacoustic flow cytometry; PAM, photoacoustic microscopy; PEG, polyethylene glycol; Pc4, phthalocyanine 4; PTC, photothermal cytometry; PVP, poly(vinylpyrrolidone); RADyK-GNC, gold nanocage conjugated with protein RADyK; RADyK-GNSt, gold nanostar conjugated with protein RADyK; RGDyK-GNSt, gold nanostar conjugated with integrin RGDyK; Route of adm., route of administration; SEM, scanning electron microscopy; SERS, surface-enhanced Raman scattering; SynCT, synchrotron-based CT; TAT, transactivator of transcription; TEM, transmission electron microscopy; Tf, transferrin; TXM, tridimensional X-ray microscopy*.

**Table 2:** Experimental models used in the included studies.

Experimental model	Examples (number of studies^a^)
*In vitro*	U87 GBM cells (6) U227 GBM cells (2) U251 GBM cells (2) GS9-6/NOTCH1 + GBM cells GBM43 cells, GL261 GBM cells 9L gliosarcoma cells, C6 glioma cells, A172 GBM cells, U373 GBM cells, BT2012036 oligodendroglioma, GLINS1 GBM stem cells B16F10 Melanoma cells MDA-MB-231 Breast cancer cells
*In vivo*	U87 GBM orthotopic xenograft (8) U87 GBM heterotopic xenograft RCAS-PDGF/N-tva transgenic mouse model of GBM GBM8401 orthotopic xenograft TS543 GBM orthotopic xenograft 9L gliosarcoma orthotopic xenograft D270 glioma orthotopic xenograft C6 glioma orthotopic xenograft F98 glioma orthotopic xenograft MDA-MB-231 breast cancer orthotopic xenograft

*^a^Number is reported only if >1*.

*GBM, glioblastoma*.

**Table 3:** Structural components of the GNPs for brain tumor diagnosis.

Structural features of GNPs		Examples (number of studies^a^)
GNP shape		Gold nanosphere (GNS) (12) Gold nanostar (GNSt) (2) Gold nanorod (GNR) (2) Gold nanocage (GNC)
GNP size		20–120 nm
GNP coating		Polyethylene glycol (PEG) (8) Silica shell (2) PEG and silica shell
GNP imaging tags	Raman tag	*Trans*-1,2-bis(4-pyridyl)-ethylene (3) IR783B *N,N*-dimethylformamide
	Fluorescent tag	Phthalocyanine 4 (Pc4) (2) Mercapto-urodecanoic acid (MUA) Cytochrome 647 (Cyto 647)
	MRI tag	Gadolinium (4)
BBB-crossing enhancers	Internal (on-surface)	RGDyK (2) transferrin (Tf) (2) Fibroblast growth factor (FGF) Low-density lipoprotein-receptor-related protein-1 (LRP-1) Transactivator of transcription (TAT) Anti-epidermal growth factor receptor antibody (anti-EGFR AB) Folate, EpCam, CD 45
	External	MR-guided focused ultrasound (MRgFUS) ultra-short pulsed laser

*^a^Number is reported only if >1*.

*GNP, gold nanoparticle*.

**Table 4:** Imaging techniques applied in conjunction with gold nanoparticles (GNPs) for brain tumor diagnosis.

Imaging resolution	Examples (number of studies^a^)
Macroscopic level	MRI (4) Synchrotron-based CT
Microscopic level	Fluorescent microscopy (4) Surface-enhanced Raman scattering microscopy (4) Photoacoustic imaging/photoacoustic microscopy (2) Photothermal cytometry Tridimensional X-ray microscopy Optical microscopy Fourier-transform infrared imaging
Subcellular level	Transmission electron microscopy (2) Multiphoton microscopy (2) Scanning electron microscopy

*^a^Number is reported only if >1*.

Sixteen studies were published between 2011 and 2017. No studies on humans were found. Eight studies were conducted *in vivo* on rodent models, three studies were performed *in vitro* on brain tumor cells, and five studies were done *in vitro* and *in vivo*. In all the studies *in vitro*, the GNPs were loaded into the brain tumor cells; while in the studies *in vivo*, the GNPs were injected intravenously into the tail vein of the rodent models with two exceptions. In one study, the GNPs were inoculated into primary breast cancer in order to detect metastatic spread to the central nervous system (32); in another study, the GNP-loaded xenograft was heterotopically injected into the subcutaneous tissue (16).

The cellular models more commonly used *in vitro* were the U87 GBM cells (six studies), the U227 GBM cells (two studies), and the U251 GBM cells (two studies). Among all the other *in vitro* only models, one study was performed on a cellular model different than glioma, namely on melanoma and breast cancer cells (32). *In vivo*, the most commonly used model was the U87 orthotopic xenograft (eight studies). A single study was performed on a cellular model different than glioma, using a breast cancer orthotopic xenograft (32). Another group used an RCAS-PDGF/N-tva transgenic mouse model of GBM (20).

The shape of GNPs was nanosphere (GNS) in 12 studies, nanostar (GNSt) in 2 studies, nanorod (GNR) in 2 studies, and nanocage (GNC) in one study. The size of GNPs ranged between 20 and 120 nm. Twelve studies used GNPs with size equal or less than 60 nm. Noticeably, the GNPs with a diameter of 120 nm crossed the BBB, after permeabilization of the brain tumor region with MRI-guided focused ultrasound (MRgFUS) (28).

Among the 13 *in vivo* studies, the GNPs were covered of polyethylene glycol (PEG) in 8 studies (namely PEGylated), the GNPs were covered with a silica shell in 2 studies, and with both in one case. Several imaging modalities used GNPs to diagnose and follow tumor growth at a macroscopic and cellular level, and to define the biodistribution of GNPs at a subcellular level. The imaging techniques are summarized in Table 5. The most commonly used imaging modalities were MRI (four studies), surface-enhanced Raman scattering (SERS) (three studies), and fluorescent microscopy (three studies). All of these techniques required specific molecules attached to the GNP surface, in order to make them detectable within the tumor cells. All the MRI-enhancing GNPs carried Gd-chelates on their surface; for fluorescent microscopy purpose, the most commonly used fluorescent tag was the phthalocyanine 4 (Pc4) (two studies); and the most commonly used SERS-active tag was the *trans*-1,2-bis(4-pyridyl)-ethylene (three studies).

**Table 5:** Reference guide for the imaging modalities used in conjunction with gold nanoparticles (GNPs).

Imaging modality	Brief description
MRI	– An oscillating magnetic field characterized by a specific resonance frequency is applied to the patient. – The hydrogen atoms generate a radiofrequency signal, which is detected by the receiving coil. – The radio signal encodes the position information by changing the main magnetic field with gradient coils. – The rate at which the excited atoms return to the equilibrium state determines the contrast between different tissues (5). – Exogenous contrast agents (i.e., gadolinium) increase the contrast between different tissues
Synchrotron-based CT	– A synchrotron source provides a high-flux, high-intensity, and monochromatic X-ray beam. – Synchrotron-based CT allows acquisition of quantitative high-resolution 3D CT images with a high signal-to-noise ratio (34)
Photoacoustic flow cytometry	– *Flow cytometry* is based on a laser source focused on cells running into a high-speed, laminar, artificial flow. – The resulting scattered light is detected by a photodetector array (35). – The main limitation of flow cytometry is the extraction of cells *ex vivo*, with potential deterioration of the physical and molecular properties of the specimen. – *Photoacoustic flow cytometry* overcomes that limitation by allowing flow cytometry *in vivo*. Conceptually, the blood and lymphatic vessels serve as tubes where cells run in a laminar flow. The photoacoustic detector captures the acoustic waves generated by laser irradiation of the cells themselves. Then, GNPs are used as intracellular contrast agents (32)
Fluorescent microscopy	– The microscope detects the light emitted by a specimen when that is irradiated with light of a specific wavelength. – Fluorescent microscopy visualizes GNPs carrying a fluorescent tag on their surface (25)
Surface-enhanced Raman scattering (SERS) microscopy	– When light impinges on a substance, a modest percentage of its energy makes the molecules of the substance shift from the baseline to the excited state, leading to the absorption of the incident photon, and emission of a scattered photon (*Raman scattered photon*) (36). – The energy exchange, and the frequency shift, between the impinging photon and the scattered photon are known as the *Raman effect*. – *Raman imaging* is defined as an optical imaging modality based on the inelastic photon scattering upon interaction with matter (37). – Since different molecules emit different Raman signal (*Raman spectrum)*, the Raman imaging is a valuable bioanalytical tool able to non-invasively differentiate molecules on the basis of their “optical fingerprint.” – In order to amplify the Raman scattering, which would be very weak in nature (36), Raman-active molecules are conjugated with metal nanoparticles. The resulting optical phenomenon is known as the SERS (38)
Tridimensional X-ray microscopy	– X-ray microscopy is a contrast imaging technology based on the difference in absorption of soft X-rays in the water window region by the carbon atoms (main element composing the living cell) and by the oxygen atoms (main element for water) (39). – After passing through the specimen, X-rays are detected by a charge-coupled device detector that forms the image
Optical microscopy	– The optical microscope (light microscope), uses visible light and a system of lenses to magnify images of small samples
Photothermal cytometry (PTC)	– The PTC is based on the temperature-dependent variation of the refractive index of a specimen irradiated with a laser. – The refractive index is detected by specific thermal lenses that allow formation of the image (32)
Fourier-transform infrared imaging (FTIR)	– FTIR is non-invasive optical fingerprinting of a biological specimen based on the analysis of light absorption different wavelength (33). – A computer infers what is the light absorption at each different wavelength, and forms an infrared spectrum of absorption of a solid, liquid, or gas
Transmission electron microscopy (TEM)	– TEM is based on irradiation of a thin specimen with electron beams of uniform current density. – The image is generated by the transmitted electrons through the specimen itself
Photoacoustic microscopy (PAM)	– PAM is an *in vivo* imaging technique that allows optical contrast detection, *via* the photoacoustic effect. – When photon beams are focused on a specimen, some of them are absorbed and their energy is converted into heat. – The heat induces a temporary pressure rise in the specimen propagating as a wideband acoustic wave. – The image is generated by an acoustic ultrasonic transducer that detects and localizes the acoustic waves in the specimen (40)
Multiphoton microscopy (MPM)	– MPM, also known as two-photon microscopy (TPM), is based on the fluorescence emission by a specimen irradiated with photon beams. – MPM is ideal for studying biological specimens because it achieves a high-imaging depth (41)
Scanning electron microscopy (SEM)	– SEM is based on detection of back-scattered and secondary electrons, when a specimen is irradiated with a high-energy beam of electrons in a raster scan pattern

**Figure 2:**
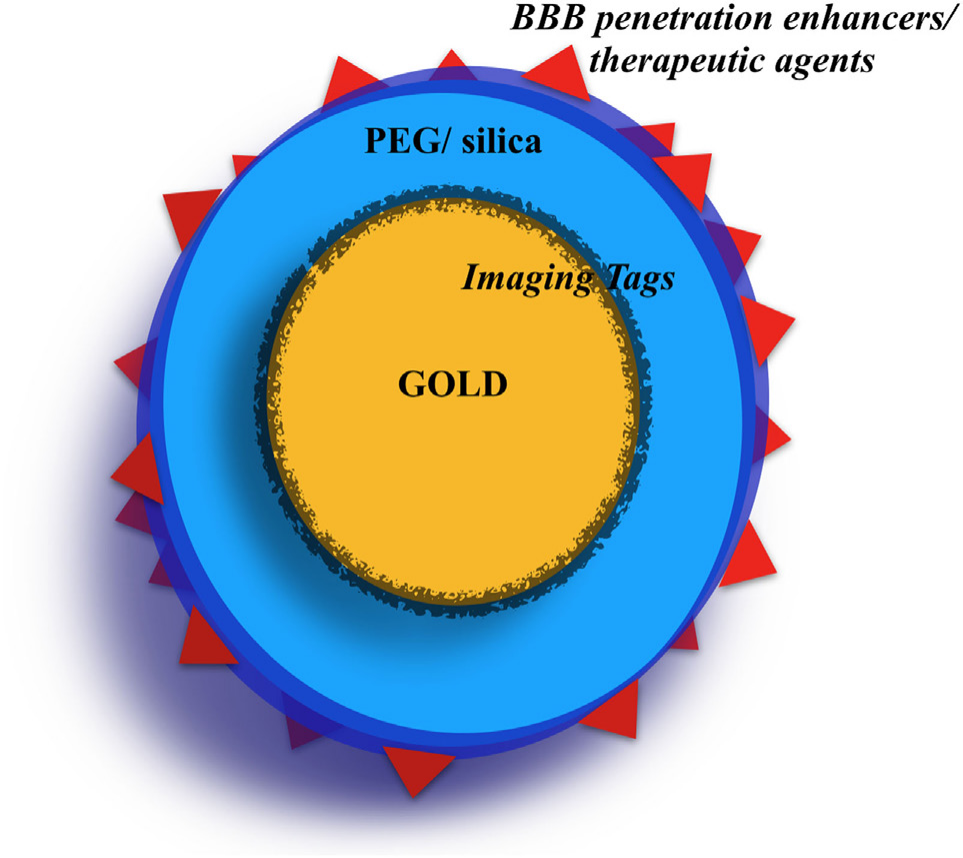
The “typical” gold nanoparticle (GNP). The most used GNPs are the nanospheres. The gold core is sometimes covered with an imaging tag (i.e., Raman tags, fluorescent tag). Then, the GNPs are often covered by a polyethylene glycol (PEG) and/or silica shell. Finally, on the GNP surface, several molecules can be conjugated serving as blood-brain barrier (BBB) crossing internal enhancers or as chemotherapy agents.

In order to increase the penetration of the GNPs through the BBB and their uptake by tumor cells, the GNPs were functionalized with different molecules on their surface (most commonly the RADyK group and the Transferrin) and/or the BBB itself was irradiated with paired-pulsed laser or MRgFUS. The GNPs were used for therapeutic purposes by the addition of a chemotherapic agent (doxorubicin) or a photosensitizer (Pc4) (Figure 2).

## Discussion

### The Structural Elements of GNPs: A Difficult Balance Between Tumor Penetration and Systemic Toxicity

The size of GNPs is critical for their physical, pharmacological, and toxic properties. The BBB is a complex barrier between the brain and the blood, which is formed by brain capillary endothelial cell tight junctions, luminal glycocalyx, basal lamina, and astrocytic foot processes. The intact BBB allows penetration of small lipophilic compounds, electroneutral compounds, and molecules under 600 Daltons. In order to use GNPs for brain imaging and/or for therapy, their size has to be tailored on the basis of the BBB restraints. Among the presented studies, the size of GNPs ranged between 20 and 120 nm, although the majority (12) had a size equal or less than 60 nm. Studies revealed that GNPs with a diameter up to 50 nm were able to pass BBB, as demonstrated by gold accumulation in the brain (42). About 0.3% of the intravenously injected GNPs with a 10 nm diameter can pass through the BBB into the brain, and this percentage remarkably drops as GNPs diameter increases (17, 42). In some studies, large GNPs (up to 120 nm) were able to cross the BBB after a temporary increase of BBB permeability, as achieved by MRgFUS (28). The size of GNPs is also critical for their toxicity. In fact, ultra-small GNPs (1.5 nm in diameter) were found to be highly cytotoxic, while approximately 10-fold larger GNPs (15 nm and above) were non-toxic at the same concentration levels (43). Thus, although there is an inverse proportion between GNP size and BBB penetration, there is not a similar correlation between size and toxicity. GNP shape can further complicate the scenario. A higher aspect ratio reduces GNPs toxicity and promotes cellular uptake (44). As an example, GNSs uptake is more efficient than GNRs uptake (44), and is less toxic (45). None of the papers on experiments performed on rodent models reported toxic reactions. Remarkably, 12 of the 13 studies performed, at least in part, using living rodent models, adopted GNSs. Other factors that might influence GNP toxicity include chemical preparation (46), high concentration, prolonged exposure (47), and the systemic route of administration (47). Nonetheless, GNPs are very biocompatible and induce minimal to no toxicity in healthy tissue (44).

The bioavailability of gold is largely dependent on the administration route. In the included papers, GNPs were injected in rodents intravenously, except when the xenograft itself was loaded with GNPs before implantation. In humans, gold preparations injected intravenously are fully absorbed within 2 h (48), while only one-fifth of the oral doses are absorbed (49). The gold diffuses to organs through the bloodstream, although the reticuloendothelial system has a remarkable affinity for the metal. Together, the liver and the bone marrow uptake 50% of injected gold (50), while the bone and skin uptake about 20% each (51). As noted above, less than 1% of injected GNPs reach the brain.

Thus, it is of paramount importance to minimize reticuloendothelial clearance of the gold, in order to maximize bioavailability for brain imaging. Various natural and synthetic polymers, including dextran (52), PEG (53), and poly(vinylpyrrolidone) (54), were employed as biocompatible coatings to prevent coagulation, promote particle monodispersion, and enhance systemic circulation of NPs. Among those, PEG was used to extensively cover the gold core of GNPs, in 9 of 13 studies performed *in vivo*. PEG is an FDA-approved biodegradable amphiphilic diblock copolymer with several advantages. First, PEG reduces GNPs clearance by the reticuloendothelial system and improves the exposure of the organs to GNPs (18); second, by reducing the reticuloendothelial clearance, PEG promotes GNPs accumulation in the brain tumor (55); third, PEG reduces potential intravascular aggregation of GNPs (aka “stealth effect”); fourth, PEG increases the retention of GNPs once there is tumor uptake (56); and fifth, the PEG coating serves as a platform for conjugation of further molecules to increase or broaden GNPs functionalities (20, 25).

### Overcoming the “Enhanced Permeability and Retention” (EPR) Effect

In 1986, Maeda et al. observed that Evans blue binding albumin selectively accumulates in tumor tissue, after endovenous injection (57). The phenomenon was named “EPR” effect. Given the aberrant and exaggerated angioarchitecture and vascular permeability of tumors, heavy (>40 kDa) molecules can diffuse into tumor interstitial space and be retained because of impaired venous and, for some organs, lymphatic drainage. The EPR phenomenon leads to remarkable accumulation of heavy (>40 kDa) molecules, such as drugs, at concentrations several folds higher than plasma. The prerequisites for the phenomenon to occur include a large molecular weight of drugs above the renal threshold, and a sufficiently high plasma concentration for a substantial amount of time (i.e., at least 6 h in mice and rat). The EPR effect still represents the pathophysiological basis for functioning of intravenously injected GNPs (19, 21). Nonetheless, the BBB of the tumor still partially limits the penetration of foreign molecules into the brain. Therefore, maximizing brain tumor uptake of GNPs is critical both for imaging and therapeutic purposes. Schematically, we divided the method for BBB penetration enhancement into two categories. The first included all molecules attached to GNPs surface (“internal enhancers”), and the second included all methods to increase BBB permeability by using an external source of energy (“external enhancers”).

#### Internal Enhancers

Internal enhancers aim to selectively increase brain tumor uptake of GNPs, making them detectable for preoperative, intraoperative, and post-operative imaging techniques. As an example, since different glioma cell lines are characterized by a variable overexpression of EGFR (58), GNPs were functionalized with a monoclonal antibody anti-EGFR (Panitumumab) promoting their internalization by tumor cells (28).

Gold nanospheres were conjugated with RGDyK, an active ligand of the integrin αvβ3, a molecular marker overexpressed in about 30% of GBMs (20). At a microscopic level, GNPs conjugated with RGDyK enhanced tumor edges, loco-regional infiltration, and satellite foci remarkably better than non-integrin specific GNPs (20).

Similarly, Gd-carrying GNPs were conjugated with a transactivator of transcription (TAT) peptide derived from HIV (TAT-GNP-Gd conjugates), in order to increase GNPs penetration through the BBB. These conjugates allowed a remarkably more intense and lasting tumor enhancement with respect to Gd alone (with a signal still detectable after 24 h) closely correlating with tumor invasion topography (41, 59). Importantly, TAT-GNP-Gd conjugates also cross the normal BBB, selectively accumulate within the most peripheral tumor cells, and are washed out from normal brain tissue (27).

Gold nanoparticles were also conjugated with multiple ligands in order to promote their uptake by cells with different molecular profiles within the same tumor mass (“molecular heterogeneity”). As an example, dual-receptor GNPs, functionalized both with FGF and transferrin, were able to cross the BBB more efficiently and accumulate more intensely in the tumor with respect to untargeted GNPs or transferrin-only targeted GNPs (25, 26).

Importantly, enhanced accumulation of functionalized GNPs into the brain did not lead to accumulation in other organs, as revealed by histological analysis of several different explanted organs (kidney, liver, heart, and lung) after injection of transferrin-functionalized GNPs (25) or after injection of dual-targeted (FGF and transferrin) GNPs (26). As a consequence, enhanced accumulation of functionalized GNPs into the tumor was not accompanied by systemic or brain toxicity. The internal enhancers were also used for GNP targeting of tumors different than gliomas, such as breast cancer or melanoma metastases (32).

Internal enhancers were used to regulate GNPs aggregation inside the tumor itself. As an example, GNPs were functionalized with either the alkyne or azide chemical group. After passing through the BBB, the acidic environment typical of solid tumors promotes PEG degradation, exposure, and interaction of the functional alkyne and azide groups, allowing GNPs aggregation. Thus, GNPs rapidly form 3D spherical nanoclusters with characteristic Raman signal and MR enhancement persisting in the brain tumor interstitium for days. Conversely, GNPs diffusing in the normal brain, do not aggregate in the neutral pH environment, and are washed out (21).

#### External Enhancers

External enhancers include all the external sources of energy promoting BBB permeabilization, such as MRgFUS (28) and laser irradiation (29). MRgFUS was used to transiently enhance the BBB permeability in order to allow very large GNPs (ranging from 50 to 120 nm) to pass through the BBB.

Next, because of the unique physical properties of GNPs, a low-power (i.e., 35 mW) laser was used to radiate a brain tumor and elicit selective extravasation of the GNPs through the BBB of the tumor, but not of healthy brain. Interestingly, this phenomenon is limited to GNPs use. Indeed, tumor irradiation with a laser of similar power did not induce extravasation of traditional contrast agents. Thus, the mechanism of tumor BBB permeation might be attributed either to direct photothermal effect or to inflammation induced by energy bust (29). The main advantage of external enhancers is that these allow over-sized GNPs to cross the BBB. The conceptual limitation of this approach is that GNPs cross the BBB only in pre-treated areas. Thus, tumor invasion boundaries should be known *a priori*. Conversely, the aim of using diagnostic GNPs is in contrast to this, namely to achieve an ultra-sensitive detection of tumor edges at a macroscopic and microscopic level. Ideally, GNPs should be engineered to diffuse freely in the brain parenchyma and to be retained only in brain tumor cells, providing an accurate “mapping” of brain tumor invasion.

### Imaging Methods: From Macrostructures to Nanostructures

Gold nanoparticles enabled imaging the development of tumors and the interaction between tumor and healthy brain tissue at different resolutions, ranging from a macroscopic level to a microscopic and to a subcellular level.

#### Macroscopic Level

The synchrotron-based head CT (SynCT) of GNPs-loaded glioma xenografts in rats, provided a 3D reconstruction of the tumor volume and shape (31, 34). This resulted in a higher spatial resolution than PET (6–8 mm) and even MRI (1 mm) (60) which is considered the “gold standard” for brain tumor diagnosis and follow-up (30). Additionally, by measuring the dilution of GNPs within subsequent tumor cells generations, SynCT allows measuring and mapping replication rates of different tumor sections (31). Unfortunately, the present approach has a limited potential for use in humans for two main reasons. First, CT imaging requires a radiation dose that might not be acceptable humans; and second, GNPs are loaded into glioma cells instead of being injected into the living animal, which does not exactly replicate the real-life scenario in humans.

On the other hand, Gd-enhanced MRI is used in daily practice. Unfortunately, Gd mainly enhances tumor portions characterized by increased BBB permeability (19, 21, 61). Thus, the main limitation of MRI is that it does not enhance the most peripheral portion of the tumor, where highly active tumor cells infiltrate healthy brain parenchyma without an extensive BBB disruption and neoangiogenesis (8). A complete tumor resection is theoretically not possible because of imprecise tumor visualization. When compared with Gd alone, GNP-Gd conjugates allowed a remarkably more intense (up to 82-fold higher intracellular Gd concentration) and more lasting enhancement of the brain tumor, with signal still detectable after 24 h. Enhancement closely correlates with the whole tumor mass (19, 21, 27). Importantly, GNP-Gd conjugates widely penetrate the normal brain parenchyma, and are quickly washed out. So, GNPs are able to cross the BBB regardless of its integrity, selectively accumulate within the tumor cells or, otherwise are removed from normal brain tissue, as expected according to the EPR effect (57). From a practical standpoint, GNP-Gd conjugates improve tumor visualization and, in the future, might improve extent of resection. Additionally, prolonged tumor enhancement might allow for improved intraoperative MRI imaging. Intraoperative MRI is mainly limited because iatrogenic BBB disruption during surgery affects Gd distribution and, as a consequence, visualization of tumor borders (62). Conversely, if GNP-Gd conjugates are injected preoperatively, tumor resection may occur in a stepwise fashion by intraoperative MRI with no need of further contrast injections, allowing for both improved diagnostic accuracy and no additional Gd-induced toxicity. Additionally, the need for quantitative and qualitative estimation of brain shift during surgery would be not practically relevant anymore, since identification of tumor edges would not depend on finding a correspondence between intraoperative and preoperative landmarks.

#### Microscopic Level

Several techniques were applied to microscopic detection of tumor spread in the brain (Table 4). From a practical viewpoint, a few of them are suitable for the clinical practice.

The photoacoustic flow cytometry (PAFC) has been used to detect breast cancer metastases *in vitro* and *in vivo* (32).

Breast cancer orthotopic xenograft bearing mice were injected with GNPs into the tumor. In the following days, a photoacoustic probe approximated to the mice cisterna magna, revealed the metastatic spread of breast cancer cells containing GNPs. PAFC detected the metastatic cells in the CSF well before macroscopic brain metastases became radiologically evident. The implementation of this technique in humans might drastically change the diagnosis of brain metastases and, potentially, the timing of treatment.

Recently, the use of fluorescent markers was found to be useful for detection of microscopic tumor foci during surgery. 5-aminolevulinic acid (5-ALA), a fluorescent tumor maker, marks tumor edges intraoperatively with good sensitivity, and spatial resolution. This has been associated with increased progression-free survival in patients affected with gliomas (63). The use of 5-ALA as tumor marker has some technical limitations. First, the natural emission of light by biological structures (autofluorescence) can result in false positives and limited depth penetration of the signal; second, fast photochemical destruction (known as “photobleaching”) limits the time of tumor enhancement (37); and third, the large spectral overlap with other fluorescent imaging agents prevents the detection of multiple targets simultaneously (known as “multiplexing”) (47). Several fluorescent tumor markers have been conjugated with GNPs in order to increase selectivity of intracellular uptake by tumor cells. As an example, fluorescent markers, such as cytochrome 647 (26) and Pc4 (28), conjugated with GNPs allowed fluorescent microscopy localizing and quantifying intracellular uptake of GNPs by tumor cells as well as by normal brain tissue and peripheral organs. Thus, the fluorescent GNPs might be used for accurate intraoperative detection of residual tumor foci. Fluorescent GNPs can be further functionalized with Raman tags (28). Although Raman imaging is not currently available in the neurosurgical practice, in experimental models, SERS microscopy revealed that GNPs delineate tumor edges, the loco-regional infiltration (corresponding to the GBM digitations) and even the satellite foci remote from the main tumor mass, strongly correlating with spatial distribution of GBM histological markers (20, 21, 28).

Importantly, Raman signal is more stable and intense with respect to fluorescent markers such as 5-ALA (64). GNPs labeled with Raman tags might be the basis for intraoperative detection of GBM cells with SERS. Additionally, the photostability of Raman-active GNPs might allow a more persistent signal emission with respect to fluorescent markers.

Nanoparticle multiplexing allows for performing multiple image modalities using the same NP. As an example, GNPs served as contrast agents for macroscopic imaging (MRI and PAFC) as well as for microscopic imaging (SERS microscopy) (19). The triple-modality NP, known as MPR (magnetic resonance–photoacoustic–Raman) NP caused stable enhancement of tumor edges and loco-regional infiltration even after 24 h post-endovenous injection, with robust correlation in the spatial distribution of the signal between the three modalities.

#### Subcellular Level

Several different techniques were employed to study the intracellular distribution of GNPs and the tumor pathophysiology. As an example, transmission electron microscopy visualizes the interaction and penetration of GNPs through the tumor membrane, the interactions of GNPs inside the tumor cells, and the ejection of GNPs from the cellular membrane (22). A correlative optical and scanning electron microscopy (SEM) technique visualized the GNPs accumulation in the brain tumor cells in experimental rodent models (24). SEM alone is not able to distinguish tumor from healthy tissue, while it allows an accurate definition of the size, shape, and structure of NPs. Thus, the GNPs can be visualized as well as be localized inside the cells (with a spatial accuracy of 10 µm), by overlaying the SEM and optical images.

Lai et al. (23) reported using X-ray microscopy to visualize the aberrant microvasculature associated with the development of brain tumors. X-ray microscopy provided the first evidence of GNPs leaking through the BBB defects of brain tumors, in contrast to the normal brain tissue unaffected by GNPs leaking. Then, Fourier-transform infrared imaging was used to characterize different patterns of tumor angioarchitecture on the basis of the specific size of fenestrations, by injecting at the same time GNPs of different sizes (33).

### “Theranostic” GNPs: The Therapeutic Side of Diagnostic GNPs

Nanoparticles can behave simultaneously as diagnostic agents, therapeutic agents, and as markers of response to chemotherapy and radiation therapy (65, 66). NPs functioning both for diagnostic and therapeutic purposes are defined as “theranostic” NPs. As an example, GNPs were conjugated with photosensitizers such as Pc4. These GNPs can be used for diagnostic purposes because of their fluorescence, as well as, for therapeutic purposes. When irradiated with a laser, Pc4 causes photosensitization, and cell death (26).

Additionally, GNPs can be used as carriers for chemotherapy agents that cannot cross the BBB under physiological conditions. As an example, doxorubicin is highly effective on glioma cells *in vitro*, and is 2,000-fold more powerful than the standard-of-care temozolomide (67). Unfortunately, doxorubicin was abandoned as a chemotherapy agent for glioma in humans because it is not able to cross the BBB. Doxorubicin conjugated with GNPs (GNP-Dox) has different pharmacokinetic properties than doxorubicin injected alone. First, after intravenous injection, almost the entire amount of GNP-Dox passed through the BBB and entered the glioma cells; second, GNP-Dox caused no toxic effect on healthy brain or on other organs such as spleen, liver, kidney, and heart; third, since the link between GNPs and Doxorubicin was achieved by an acid-labile hydrazone group, the GNP-Dox could selectively release doxorubicin within the acidic environment of the lysosomes (pH = 4.5–6.0), leading to glioma cell death. As a consequence, a highly selective and highly concentrated topical chemotherapy is achieved, with no adverse effects. The use of GNPs as chemotherapy carriers might open the door to reconsider other agents that are currently not deemed efficacious for brain tumor treatment.

## Limitations

At the moment, the interaction of the structural features of GNPs with their physical, biological, and toxic properties is not completely clear. A more detailed understanding would be crucial to maximize brain tumor uptake and to minimize the potential toxic effects on humans. Additionally, a quantitative and statistical comparison between the results of the included studies was not possible, because of the heterogeneity of the biological models, as well as, of the structural and biological features of the used GNPs.

Importantly, our review did not provide any evidence of ongoing or completed clinical trials. Thus, all the conclusions regarding the potential applications of GNPs for brain tumor diagnosis and treatment should be considered field of ongoing research. A clinical application is still not available. Nonetheless, ample experience in the use of gold for medical purposes (i.e., rheumatoid arthritis) makes GNPs promising agents for brain tumor diagnosis and treatment in humans.

## Conclusion

**Figure 3:**
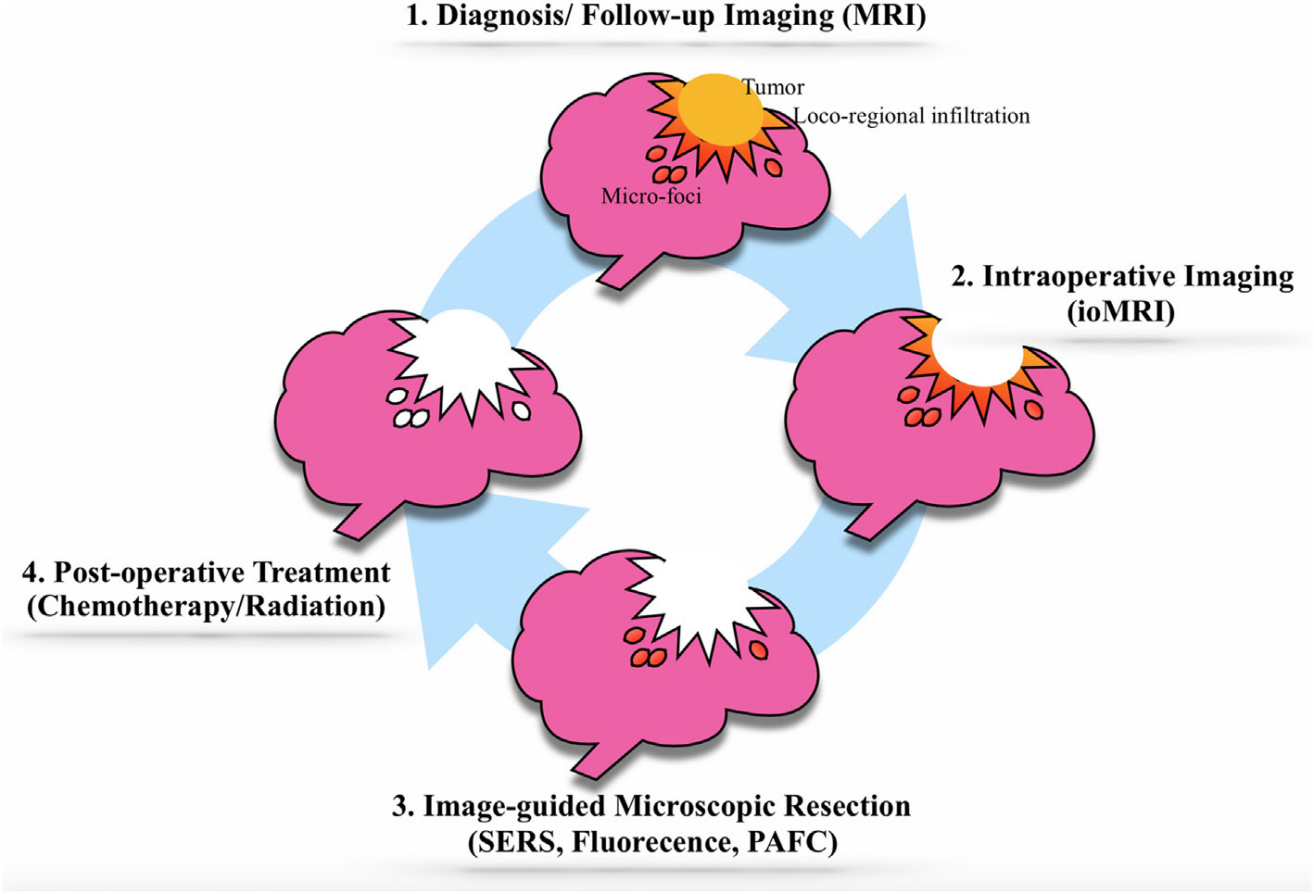
The theranostic cycle. (1) The brain tumor diagnosis is achieved by advanced brain magnetic resonance imaging (MRI) with gadolinium-conjugated gold nanoparticles (GNPs). (2) After the usual surgical debulking of the tumor mass, the loco-regional invasion is identified and removed in two steps: a macroscopic phase [using intraoperative MRI (ioMRI)] and (3) a microscopic phase involving GNPs suitable for Raman imaging, fluorescent imaging, photoacoustic imaging, photoacoustic flow cytometry. (4) The GNPs can be loaded with therapeutic agents, such as chemotherapy agents, that can target and destroy potential tumor residuals. If the tumor should recur, the cycle may be repeated.

Gold nanoparticles are highly selective contrast agents for preoperative, intraoperative (with macroscopic and microscopic resolution), and post-operative imaging of primary and metastatic brain tumors. In comparison with Gd alone, GNPs can persist in the tumor mass for hours and potentially days after a single injection. The selectivity of GNPs for brain tumor cells, their prolonged retention in the tumor itself, as well as their effectiveness as therapeutic agents provides the theoretical basis for the “theranostic cycle” of GNPs (Figure 3). Injected GNPs allow for accurate delineation of the tumor mass and its loco-regional invasion, as visualized by MRI. Once tumor debulking has been completed, intraoperative imaging might allow for detailed macroscopic (by intraoperative MRI) and microscopic (by Raman imaging and fluorescent imaging) detection and removal of loco-regional tumor invasion. Next, the most remote satellite brain tumor foci can be treated with local focused chemotherapy. Should the tumor recur, a new “theranostic cycle” can be repeated because of the minimal toxicity of GNPs *in vivo*.

## Nomenclature

**Table:** 

Anti-EGFR Ab	Anti-epidermal growth factor receptor antibody
BBB	Blood-brain barrier
Cyto647	Cytochrome 647
EPR effect	Enhanced permeability and retention effect
FGF	Fibroblast growth factor
Fluo	Fluorescent imaging
FTIR	Fourier-transform infrared imaging
Gd	Gadolinium
GNC	Gold nanocage
GNP	Gold nanoparticle
GNP-Dox	GNP conjugated with doxorubicin
GNR	Gold nanorod
GNS	Gold nanosphere
GNSt	Gold nanostar
LRP-1	Low-density lipoprotein-receptor-related protein-1
MPM	Multiphoton microscopy
MPR	Magnetic resonance imaging–photoacoustic imaging–Raman imaging
MRgFUS	MRI-guided focused ultrasound
MRI	Magnetic resonance imaging
MUA	Mercapto-urodecanoic acid
NP	Nanoparticle
PAFC	Photoacoustic imaging
PAM	Photoacoustic microscopy
PEG	Polyethylene glycol
PFS	Progression-free survival
Pt4	Phthalocyanine 4
PTC	Photothermal cytometry
PVP	Poly(vinylpyrrolidone)
RADyK-GNC	Gold nanocage conjugated with protein RADyK
RADyK-GNSt	Gold nanostar conjugated with protein RADyK
RGDyK-GNSt	Gold nanostar conjugated with integrin RGDyK
SEM	Scanning electron microscopy
SERS	Surface-enhanced Raman scattering
SynCT	Synchrotron-based CT
TAT	Transactivator of transcription
TEM	Transmission electron microscopy
Tf	Transferrin
TXM	Tridimensional X-ray microscopy
5-ALA	5-aminolevulinic acid

## Author Contributions

AM and SC concept and design, AM and NC data collection and tables and figures, AM, JR, SC, and MS analysis of data, AM and JR drafting of the paper, JR and SC critical review of the manuscript, All the authors final approval.

## Conflict of Interest Statement

The authors declare that the research was conducted in the absence of any commercial or financial relationships that could be construed as a potential conflict of interest.
